# Phenomenological Modeling of Formic Acid Fractionation of Sugarcane Bagasse by Integration of Operation Parameters as an Extended Combined Severity Factor

**DOI:** 10.3390/molecules26092753

**Published:** 2021-05-07

**Authors:** Xiaogang Chang, Jingzhi Zhang, Ruchun Wu, Xuebing Zhao

**Affiliations:** 1 Guangxi Key Laboratory of Chemistry and Engineering of Forest Products, School of Chemistry and Chemical Engineering, Guangxi University for Nationalities, Nanning 530006, China; changxg18@163.com; 2 Key Laboratory of Industrial Biocatalysis, Ministry of Education; Tsinghua University, Beijing 100084, China; zjz5599@163.com; 3 Institute of Applied Chemistry, Department of Chemical Engineering, Tsinghua University, Beijing 100804, China

**Keywords:** lignocellulosic biomass, formic acid fractionation, phenomenological modeling, extended combined severity factor

## Abstract

In order to more conveniently simulate and optimize the solubilization of sugarcane bagasse components during formic acid (FA) fractionation, an extended combined severity factor (*CSF*_ext_) was defined to integrate various operation parameters as a single factor. Two phenomenological models based on Arrhenius and Logistic equations were further used to describe the phenomenological kinetics. Different data-processing methods were compared to fit the severity parameters and model constants. Both Arrhenius-based and Logistic-based models show satisfying fitting results, though the values of Arrhenius-based *CSF*_ext_ (A-*CSF*_ext_) and Logistic-based *CSF*_ext_ (L-*CSF*_ext_) were somewhat different under the same fractionation condition. The solubilization of biomass components increased with *CSF*_ext_, but two distinct stages could be observed with inflection points at A-*CSF*_ext_ of 42 or L-*CSF*_ext_ of 43, corresponding to bulk and residual solubilization stages, respectively. For the enzymatic hydrolysis of cellulosic solids, the highest initial enzymatic glucan conversion (EGC@6h) was obtained at A-*CSF*_ext_ of 39–40 or A-*CSF*_ext_ of 40–41; however, for a long hydrolysis period (72 h), relatively high glucan conversion (EGC@72h) was observed at A-*CSF*_ext_ of 42–43 or A-*CSF*_ext_ of 43–44. Post-treatment for deformylation with a small amount of lime could help to recover the cellulose digestibility.

## 1. Introduction

Currently, in the face of multiple challenges such as oil shortage, climate change and environmental pollution, a reduction in the dependence on traditional fossil energy sources has aroused increasing attention. Therefore, the utilization of green and renewable resources to produce fuel is an important direction for low-carbon development [1]. Lignocellulosic biomass, with a huge yield and low price, has been considered as one of the most important renewable feedstocks for producing biofuels, such as ethanol, which has been commercially used as a substitute for fossil gasoline [2,3]. However, during the bioconversion of lignocellulose to bioethanol, pretreatment is a key step to improve the cellulose hydrolyzability for efficient release of sugars from the plant cell wall. This was mainly due to the biomass recalcitrance constructed by cell wall components, mainly hemicellulose and lignin, and their complicated interactions [4]. Therefore, removing hemicelluloses and lignin can greatly increase cellulose accessibility by exposing the cellulose surface [5]. Various pretreatment methods have been developed to overcome biomass recalcitrance, among which organosolv pretreatment can provide a unique way of achieving both the effective exposure of cellulose surface to increase its digestibility and fractionation of biomass to obtain cellulose-rich solids, hemicelluloses and high-purity lignin [6,7]. Formic acid (FA) has been considered as a good solvent for the fractionation of lignocellulosic biomass, because of its acidity and good ability to dissolve lignin [8]. Good fractionation of biomass components was achieved by FA treatment of various lignocellulose, such as sugarcane bagasse [9,10], wheat straw [11], corn stover [12] and even woody biomass [13]. However, flexible control of the operation parameters of the fractionation process based on kinetics is important in order to maximize the efficiency. Nevertheless, unfortunately, chemical pretreatment of biomass is a typical complex reaction system involving liquid–solid two-phase or gas–liquid–solid three-phase reactions, while rigorous kinetic modeling based on a set of elementary steps is not possible. Therefore, modeling such complex systems is essentially phenomenological. Thus, the estimated kinetic parameters are functions of the ranges of experimental conditions used [14]. For example, a severity factor (*SF*, also termed a severity ordinate, shown as Equation (1) was introduced to integrate the effects of several factors on an evaluation of the biomass pretreatment process, especially for dilute acid prehydrolysis [14,15]. *SF* is also more convenient in industrial applications for such complex reactions because it combines the effects of the different operational variables into a single parameter

(1) $$SF=\log R_{0}=\log[t\times \exp(\frac{T-T_{\mathrm{ref}}}{14.75})]$$

where *R_C_*_0_ is the severity parameter; *t* is pretreatment time (min); *T* is pretreatment temperature (°C); and *T*_ref_ is reference temperature and usually selected as 100 °C. However, in *SF*, the contribution of acidity is not considered. To include the effect of acidity on pretreatment severity, a combined severity factor (*CSF*) was further defined, as shown in Equation (2) [16]

(2) $$CSF=\log R_{C}{}_{0}=\log[t\times \exp(\frac{T-T_{\mathrm{ref}}}{14.75})-\mathrm{pH}]$$

where *R_C_*_0_ is the combined severity parameter. *CSF* has been widely and directly used to investigate the reaction conditions for different pretreatments or fractionations of various biomass feedstocks, including dilute acid pre-hydrolysis [17,18], steam explosion [19,20] and organosolv processes [21,22]. However, for organosolv fractionation process, the solvent’s contribution to severity should be also considered. As was found by Zhao and Liu [23], during the acid-catalyzed acetic acid delignification of sugarcane bagasse, the acetic acid concentration affected the solubility parameter of the solution and the ability to form hydrogen bonds with lignin fragments, which significantly affected the observed kinetic behavior of delignification. To further involve the contribution of solvent to the organosolv pretreatment of lignocellulosic biomass, our group defined an extended combined severity factor (*CSF*_ext_) (Equation (3)) for the phenomenological modeling of FA fractionating pretreatment of wheat straw, and the model parameters were fitted by experimental data of xylan and lignin solubilization [24]. The developed model showed a satisfying prediction of xylan solubilization as well as delignification under different conditions.

(3) $$CSF_{\mathrm{ext}}=(\frac{T-T_{f}}{\omega })+\ln t+m\ln C_{\mathrm{sol}}^{}+n\ln C_{\mathrm{cat}}^{}$$

*CSF*_ext_ may provide a more general definition of the severity factor and can be applied as an integrated parameter, involving the contribution of temperature, time, solvent and catalyst concentrations, for evaluating the pretreatment process. Therefore, the objective of this work is to study whether the *CSF*_ext_ can be applied for the phenomenological modeling of the FA fractionation of another biomass feedstock, sugarcane bagasse. Notably, how to fit the model parameters based on experimental data was further investigated. The finding of this work may provide useful information to guide the phenomenological modeling of the biomass pretreatment process by integrating the contribution of different factors as a single factor to optimize the operation parameters.

## 2. Results

### 2.1. Definition of Extended Combined Severity Factor and Phenomenological Modeling

In the definition of *CSF*_ext_ (Equation (3)), the total reaction severity is calculated by sum of the “contribution” of each operation factor. Corresponding constants are involved, which reflect the relative significance of each factor. The lowest temperature used in the experiments is usually selected as the *T*_ref_. For example, for dilute acid pretreatment, *T*_ref_ is usually selected as 100 °C, as initially defined by Chum et al. [16], while, for FA fractionating pretreatment of wheat straw, 70 °C was selected as the *T*_ref_ [24]. *ω* is an experiment-determined severity parameter related to the reduced activation energy [16]; *t* is reaction time with a unit of minute; *C*_sol_ and *C*_cat_ are solvent and catalyst concentrations with units of mol/L, respectively; *m* and *n* are the observed reaction orders with respect to solvent and catalyst concentrations, respectively. In the present work, no external mineral acid catalyst was used, because FA played the role of both catalyst (since FA can dissociate H^+^) and lignin solvent; therefore, the contribution of catalyst (H^+^) can be incorporated into the effect of solvent concentration. Thus, the corresponding extended combined severity factor (*CSF*_ext_) for FA pretreatment of sugarcane bagasse in this work can be simplified as

(4) $$CSF_{\mathrm{ext}}=(\frac{T-T_{f}}{\omega })+m\ln C_{\mathrm{FA}}+\ln t$$

where *C*_FA_ is FA concentration in mol/L. The determination of the constants *ω* and *m* is thus the first step in the phenomenological modeling of FA fractionation of sugarcane bagasse. However, different results may be obtained when experimental data on the solubilization of different polymeric components of biomass are used for fitting, because these components showed different *ω* and *m* reaction behaviors in response to the reaction conditions. For instance, in dilute acid hydrolysis, xylan is the primary component removed, and thus it is better to use the experimental data of xlyan removal (or xylose yield) to determine *ω* [24]. In the FA fractionation process, both xylan and lignin are significantly removed, and, thus, data on xylan and lignin solubilization should be used to determine the constants. A comparison of the different data-processing methods used to fit these constants was the major objective of this work.

For phenomenological modeling with *CSF*_ext_, at least two models have been proposed, as reported by Dong et al. [24], namely, models based on the Arrhenius equation and modified Logistic equation. The Arrhenius equation-based model assumes that the solubilization of biomass components follows a typical homogeneous first-order mechanism, while the Logistic equation-based model employs the assumption that the rate of the biomass component solubilization is a first-order reaction with respect to the degree of solubilization itself, as well as the un-removed fraction. By defining the main objective function, the degree of polymeric component solubilization (*α*) as the weight ratio of solubilized fraction to the initial part of the component, the model based on Arrhenius equation assumes that ln[−ln(1 − *α*)] has a phenomenologically linear relationship with *CSF*_ext_, namely

(5) $$\alpha =1-\exp[-\exp(aCSF_{\mathrm{ext}}+b)]$$

where *a* and *b* are model parameters. The model based on the modified Logistic equation assumes that ln(1/(1 − *α*) − 1) has a linear relationship with *CSF*_ext_, namely

(6) $$\alpha =1-\frac{1}{1+\exp(qCSF_{\mathrm{ext}}+c)}$$

where *q* and *c* are corresponding model parameters. Therefore, the severity constants *ω* and *m* in *CSF*_ext_, and the linear relationship parameters *a* and *b*, *q* and *c* can be determined by a multiple linear regression of experimental data at different pretreatment temperatures, times and FA concentration levels.

### 2.2. Determination of Severity Constants and Model Parameters

**Figure 1:**
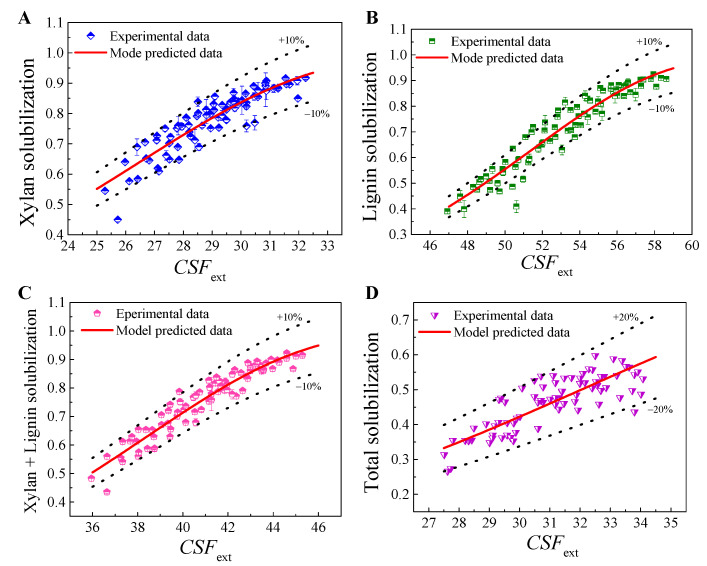
Fitting of severity constants and model parameters with Arrhenius equation-based model. (**A**) with xylan solubilization data for fitting; (**B**) with lignin solubilization data for fitting; (**C**) with xylan plus lignin solubilization data for fitting; and (**D**) with total biomass solubilization data for fitting.

**Figure 2:**
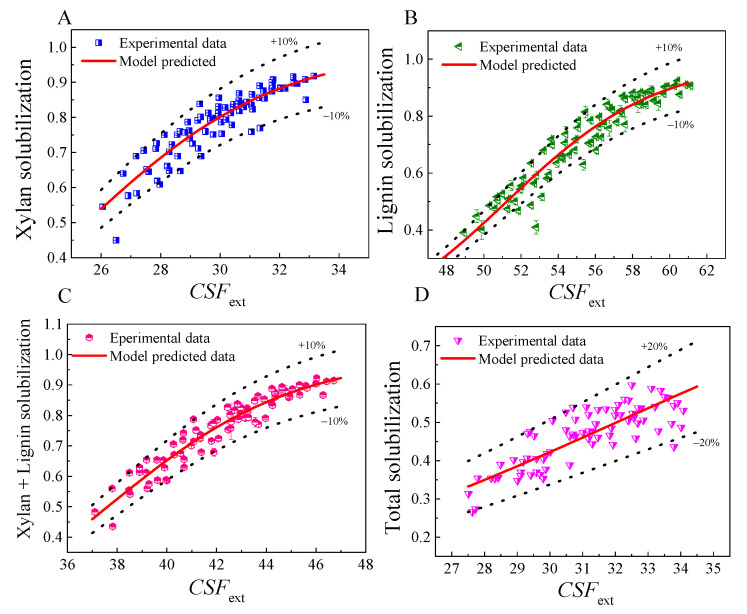
Fitting of severity constants and model parameters with Logistic equation-based model. (**A**) with xylan solubilization data for fitting; (**B**) with lignin solubilization data for fitting; (**C**) with xylan plus lignin solubilization data for fitting; (**D**) with total biomass solubilization data for fitting.

During FA pretreatment, a significant removal of xylan and lignin was observed. A portion of cellulose can also be solubilized. Therefore, the severity constants should mainly be determined by the experimental data of xylan and lignin solubilization under different pretreatment conditions. However, even so, there are still different methods for data processing. For example, the severity constants, *ω* and *m*, can be separately fitted by experimental data of xylan and lignin solubilization, and averages were then used as the final determined values, as performed by Dong et al. [24]. When the fitted values of *ω* and *m* were similar for xylan solubilization and delignification, this data-processing method make sense. However, if the fitted values are much different, using the average as the final value may lead to large errors. Therefore, more data-processing methods should be further studied and compared. In this work, the severity constants and model parameters were separately fitted with experimental data on solubilization of xylan, lignin, xylan plus lignin fraction, and total biomass, as shown in Table 1. Corresponding plots of experimental data and model-predicted data for xylan and lignin solubilization with respect to *CSF*_ext_ are shown in Figure 1 and Figure 2. 

The results illustrate that both models showed a good degree of fitting to the experiment-determined data with high determination coefficients (*R*^2^ > 0.83) for xylan solubilization, delignification (*R*^2^ > 0.91) and xylan plus lignin solubilization (*R*^2^ > 0.91). The deviation between predicted data and experimental data was generally in the range of ±10–However, for total biomass solubilization, *R*^2^ was only about 0.60 and the deviation was 20%. This was because the kinetics of solubilization of xylan and hemicellulose during FA fractionation were similar, but the solubilization of glucan showed very different kinetic behavior; thus, a high deviation could be observed when the data of total biomass solubilization were used for fitting. Nevertheless, the *F* values for each data-processing method were very high, with correspondingly very low *P* values, indicating that these models (Equation (5) and Equation (6)) were statistically significant in describing the phenomenological kinetics of solubilization of the biomass component during FA pretreatment. Both Logistic equation-based and Arrhenius based models showed a very similar degree of fitting. Table 1 also shows that the value of *ω* was not a fixed constant and varied depending on the data-processing methods. The value of *m* was high, indicating that FA concentration had a very significant effect on the fractionation, and thus made a significant contribution to *CSF*_ext_. Figure 1 and Table 1 also suggested that the data of xylan plus lignin solubilization, namely by considering the xylan and lignin as a soluble fraction of biomass, were preferred to fit the model parameters. Thus, the *CSF*_ext_ for FA fractionation of sugarcane bagasse can be calculated according to pretreatment conditions as follows.

Based on Arrhenius equation (A-*CSF*_ext_)

(7) $$A\text{-}CSF_{\mathrm{ext}}=(\frac{T-70}{16.795})+12.165\ln C_{\mathrm{FA}}+\ln t$$

Based on Logistic equation (L-*CSF*_ext_)

(8) $$L\text{-}CSF_{\mathrm{ext}}=(\frac{T-70}{16.32})+12.67\ln C_{\mathrm{FA}}+\ln t$$

### 2.3. Application of the Extended Severity Factor to Evaluate the Pretreatment Process

#### 2.3.1. Use of *CSF*_ext_ as an Integrated Parameter to Correlate Operation Condition with Solubilization of Biomass Components

**Figure 3:**
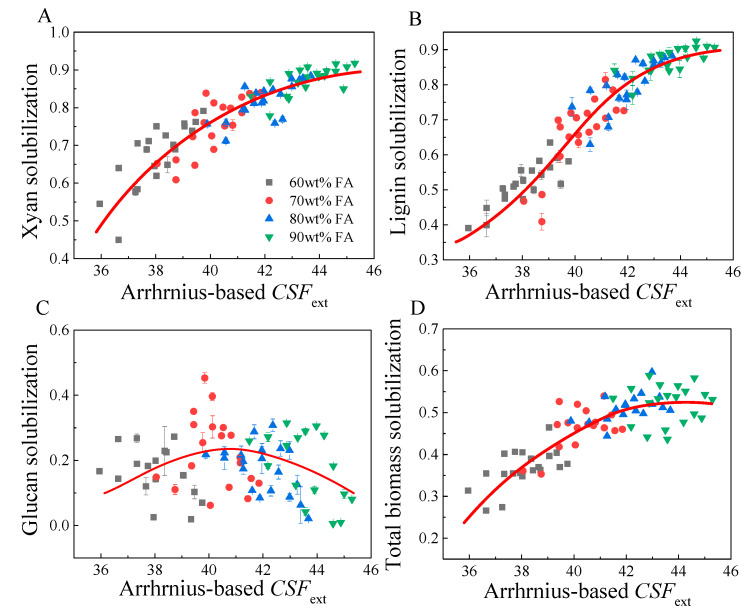
Plots of Arrhenius-based *CSF*_ext_ with total xylan solubilization (**A**), lignin solubilization (**B**), glucan solubilization (**C**) and total biomass solubilization (**D**).

**Figure 4:**
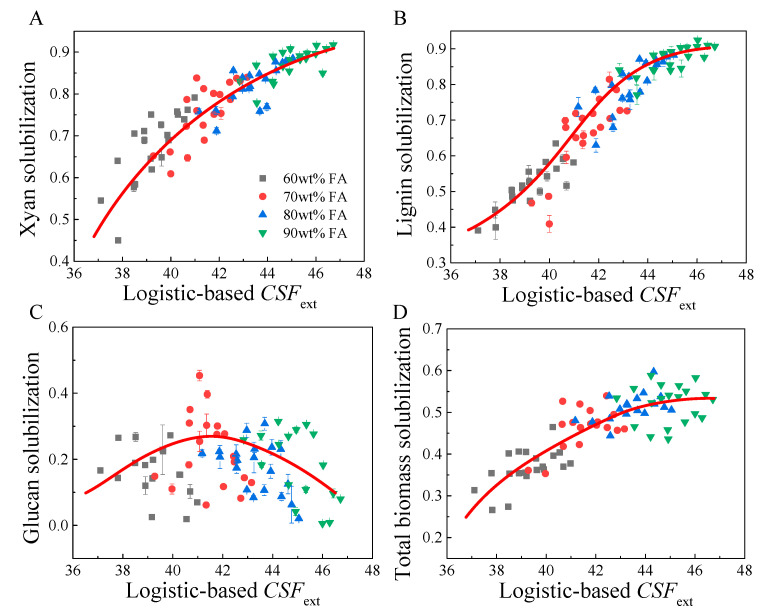
Plots of Logistic-based *CSF*_ext_ with total xylan solubilization (**A**), lignin solubilization (**B**), glucan solubilization (**C**) and total biomass solubilization (**D**).

Plots of the total biomass, glucan, xylan and lignin solubilizations versus Arrhenius-based (Equation (7)) *CSF*_ext_ and Logistic-based *CSF*_ext_ (Equation (8)) are shown in Figure 3 and Figure 4, respectively. A very similar tendency was observed for both plots of biomass component solubilization with Arrhenius-based and Logistic-based *CSF*_ext_. As shown in Figure 3A and Figure 4A, xylan solubilization increased significantly in the range of 36–42 for Arrhenius-based *CSF*_ext_ or 38–44 for Logistic-based *CSF*_ext_, but with a diminishing rate at a *CSF*_ext_ higher than 42 (Arrhenius-based) or 44 (Logistic-based). For delignification, lignin solubilization changed in the range of 0.4–1.0, when *CSF*_ext_ increased from 36 to 48. However, inflection points can be observed at an Arrhenius-based *CSF*_ext_ of 43 or Logistic-based *CSF*_ext_ of 44. This was mainly because the residual lignin was difficult to remove even though the reaction severity was further enhanced, which could also be explained by the marginal effect. For glucan solubilization, the datapoints were very discrete, but an implicit tendency could be observed, where glucan solubilization slightly increased and then decreased. The highest glucan solubilization seemed to appear at a *CSF*_ext_ of 40–43. Higher *CSF*_ext_ seemed to oppositely decrease glucan solubilization. This was probably because the high concentration of FA could significantly contribute to the *CSF*_ext_ but less water was present in the system, thus reducing the hydrolysis of cellulose. However, the glucan solubilization was relatively small, and larger errors could occur in the experiments. For total biomass solubilization, a similar tendency to that of xylan solubilization was found. A relatively rapid increase in total biomass solubilization was observed at an Arrhenius-based *CSF*_ext_ of 36–42 or Logistic-based *CSF*_ext_ of 38–44. This was primarily due to the solubilization of xylan and lignin. A further increase in *CSF*_ext_ just slightly increased the total biomass solubilization, which was mainly due to the difficulties in the solubilization of cellulose and residual xylan and lignin. However, the objective of FA fractionation is to liberate cellulose fiber from the cell wall matrix by removing hemicelluloses and lignin, and cellulose should be recovered as a solid phase. Thus the reaction was best performed at an Arrhenius-based *CSF*_ext_ higher than 42 or Logistic-based *CSF*_ext_ higher than 43, particularly with a high FA concentration, for example, 90% FA.

#### 2.3.2. Evaluation of Enzymatic Digestibility of Cellulosic Solids

**Figure 5:**
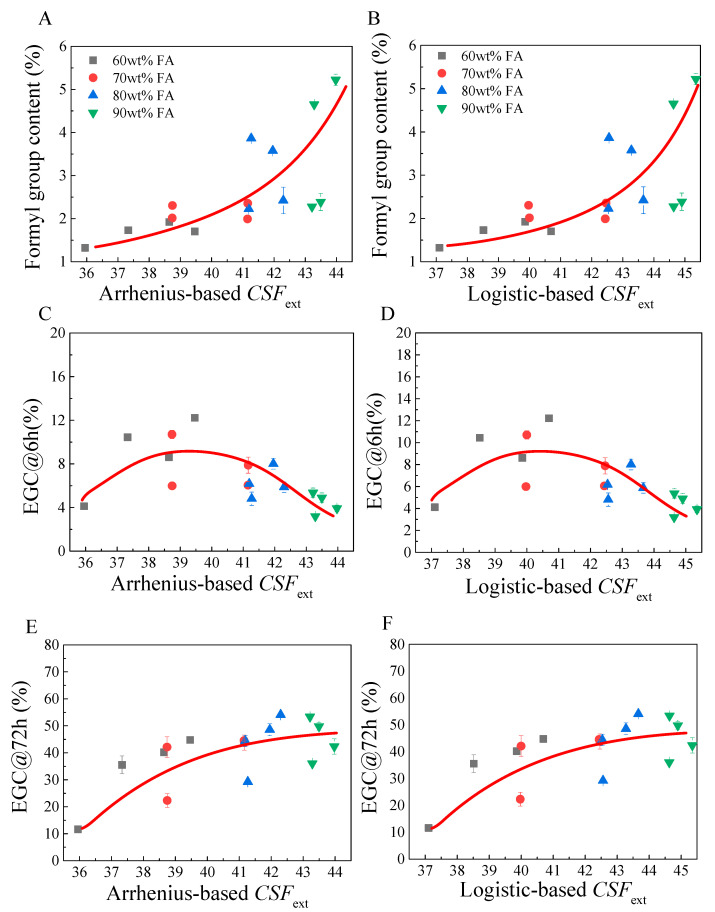
Plots of *CSF*_ext_ with formyl group content (FC) (**A**,**B**) and enzymatic glucan conversion (**C**–**F**) of pretreated cellulosic solid.

The obtained cellulosic solid may have many applications. One of its uses is to produce glucose by enzymatic hydrolysis for the further production of biofuels such as ethanol. Therefore, experiments were further performed to investigate the effects FA fractionation condition on cellulose digestibility. An orthogonal experimental design (L_16_(3^5^)) was used to arrange the experimental runs with solid yield (SY), glucan content (GC), xylan content (XC), lignin content (LC), xylan removal (XR), degree of delignification (DD), formyl group content (FC), enzymatic glucan conversion (EGC) at 6h and 72h as the objective variables. Corresponding *CSF*_ext_ were also calculated according to the operation parameters. The results are listed in Table 2. The *CSF*_ext_ was in a wide range of 35–46, and the SY, XR and DD were in the range of 45–90%, covering the typical results of FA fractionation. Because cellulose contains three hydroxyl groups in each glucose unit, formylation can take place during FA fractionation, leading to an increase in FC, which may reduce the molecular recognition of cellulases for hydrolysis [25]. Plots of *CSF*_ext_ with FC of cellulosic solids are shown in Figure 5A,B. It is clear that FC continuously increased with *CSF*_ext_, but FC was just slightly increased with *CSF*_ext_ at A-*CSF*_ext_ range of 36–41 or L-*CSF*_ext_ range of 37–42. However, a significant increase in FC was observed at an A-*CSF*_ext_ higher than 41 or an L-*CSF*_ext_ higher than 42. This was primarily because, at this inflection point, a large portion of hemicelluloses and lignin was removed, leading to a significant exposure of cellulose, resulting in an increased degree of formylation. For enzymatic hydrolysis of cellulosic solid, EGC@6h and EGC@72h were compared, which represents the initial rate of enzymatic hydrolysis and final degree of enzymatic hydrolysis of glucan, respectively. Plots of EGC@6h and EGC@72h with A-*CSF*_ext_ and L-*CSF*_ext_ are shown in Figure 4. It was observed that EGC@6h generally increased with A-*CSF*_ext_ and L-*CSF*_ext_ to achieve the maximal at the A-*CSF*_ext_ of 39–40 or A-*CSF*_ext_ of 40–41 (Figure 5C,D). Further increasing *CSF*_ext_ oppositely reduced EGC@6h. This was because, at a low *CSF*_ext_, the removal of hemicelluloses and lignin was not high enough, and thus the exposure of cellulose was not good enough. However, at high *CSF*_ext_, the formylation of cellulose became significant, thus reducing the productive adsorption of cellulases for initiating the hydrolysis process. Nevertheless, EGC@72h seemed to be continually increasing with *CSF*_ext_, but no further apparent increase was observed at an A-*CSF*_ext_ higher than 41 or L-*CSF*_ext_ higher than 42(Figure 5E,F). This phenomenon was highly in accordance with that observed for FC. The substitution of cellulose hydroxyl group by the formyl group may lead to interference for the recognition of cellulase enzymes to cellulose substrates by inhibiting the formation of hydrogen bonds (productive binding) between cellulose and the catalytic domain of cellulases [25]. The diameter of a cellulose chain might also be enlarged by formyl group substitution, which may reduce its chance entering the tunnel or groove in the catalytic domain of cellobiohydrolase, the most important cellulase component [26].

The results shown in Table 2 and Figure 5 thus suggested that the EGC of cellulosic solid obtained by FA fractionation was affected by operation condition, namely *CSF*_ext_, by a complicated mechanism. Increasing *CSF*_ext_ resulted in a higher degree of removal of hemicelluloses and lignin, which were beneficial to expose cellulose surface for enzymatic hydrolysis. Notably, delignification has been found to greatly increase cellulose accessibility [27]. FA fractionation works like a chemical pulping process that liberates cellulose fibers by delignification. Once DD reaches a certain point, which is known as the point of fiber liberation or defibration point, the cellulose fiber becomes liberated, with little or no mechanical action [28]. After cellulose fiber is liberated, its accessibility is greatly improved [29].Therefore, a generally higher EGC can be obtained at a higher DD. However, because the formylation reaction became significant once cellulose fibers were liberated, the EGC of formylated cellulose was reduced. Therefore, apparently, the highest EGC was not obtained at the points with the highest degree of delignification. For example, the experimental results shown in Table 2 demonstrated that the highest DD (89.2 ± 1.4%) was obtained by Run #8 at A-*CSF*_ext_ of 43.97 or L-*CSF*_ext_ of 45.35, but EGC@6h and EGC@72h were just 3.9 ± 0.3% and 42.3 ± 2.9%, respectively. Thus, the above results indicated that, in order to recover the cellulose digestibility, deformylation was necessary.

In a practical process, an efficient way of eliminating the negative effects of the formyl group is alkaline saponification. For example, lime or ammonia can be used to remove the introduced formyl group. When ammonia is used, the formed ammonia salts can be utilized by yeast as a nitrogen source in the subsequent fermentation process. However, lime is usually much cheaper than ammonia. In the present work, the cellulosic solid obtained by FA fractionation performed at A-*CSF*_ext_ of ~43 or L-*CSF*_ext_ of ~44 (80% FA, 105 °C, 0.5 h, as run # 15 in Table 2 and 90% FA, 105 °C, 0.25h as run # 16 Table 2) were further post-treated with 4 wt% lime based on the initial dry sugarcane bagasse solid. The chemical components and EGC of de-formylated solids are shown in Table 3. Most of the formyl groups could be removed, with the FC being reduced to less than 0.5%. Compared with the samples without deformylation, the efficiency of long-time enzymatic digestion (EGC@72h) was significantly enhanced to 72–75%, while the initial rate of enzymatic hydrolysis (EGC@6h) were even more significantly increased, from less than 6% of formylated substrates to higher than 40% of deformylated samples (Table 3), indicating the efficiency of deformylation for recovering the cellulose digestibility of cellulosic solids obtained by FA fractionation. In the experiments, we even found that, for a shorter hydrolysis, for example, 3h, the EGC of deformylated substrates could be as high as 30–35%. These results corroborated that removing the formyl group promotes the recognition of cellulose molecules, thus improving the productive adsorption of cellulase enzymes. 

Therefore, the above results indicated that *CSF*_ext_ could be applied as an integrated parameter to evaluate the phenomenological kinetics of FA fractionation of sugarcane bagasse. Compared with traditional pseudo-homogenous kinetics, *CSF*_ext_-based phenomenological modeling is simpler, with acceptable accuracy, but even more applicable in a practical industrial process to guide the selection of pretreatment conditions.

## 3. Discussion

The fractionation of lignocellulosic biomass with organosolv solvents takes place in a complicated system, in which mass transfer including external and internal diffusion of solvents and catalysts, as well as solubilized biomass components, and reactions of polysaccharides and lignin are involved. For such a complicated system, rigorous kinetic modeling is impossible. However, phenomenological modeling is usually useful for optimizing the operation conditions. *CSF*_ext_ is an integrated parameter, considering the contribution of different factors such as temperature, time and solvent (FA) concentration. Such an integration is reasonable, because, to achieve similar fractionation efficiency, the reaction can be performed at a higher temperature for a shorter time or lower temperature for a longer time. However, the “contribution” of each factor to the reaction severity is different, so that an adjustable constant is introduced, corresponding to each factor. Therefore, fitting of the constants in *CSF*_ext_ was the prerequisite step for phenomenological modeling. The relationship between fractionation efficiency, for example, xylan solubilization or degree of delignification and *CSF*_ext_ definitely affects the values of fitted constants. Mathematically, many equations can be used to fit the experimental data. Nevertheless, it is better for the relationship to be deduced from a classic kinetic model, such as the Arrhenius equation. In this work, the solubilization of biomass components was considered as a linear increase with *CSF*_ext,_ which was also applied in previous works [16,24]. An Arrhenius-based model that can be developed from a first-order pseudo-homogenous kinetic model was used to correlate the relationship between different factors and xylan or lignin solubilization. Moreover, a phenomenological model developed based on modified Logistic equation [24] was also used for comparison. Both models showed similar accuracy to fit the experimental data. However, since xylan, lignin and cellulose show different solulibization behaviors in FA fractionation, the fitted constants are different when different data-processing methods are used for fitting. As indicated in Table 1, differently fitted severity parameters and kinetic constants indeed could be obtained when data on the solubilization of xylan, lignin, xylan plus lignin and total biomass were used. During FA fractionation, xylan and lignin were the major components solubilized from solid phase to liquid phase. Thus, by considering xylan plus lignin as a “pseudo-soluble” fraction, the fitted constants seemed to be the most suitable and compromised. The results indicated that such a phenomenological model could describe the effects of different factors on the objective functions, such as xylan or lignin solubilization, with satisfying accuracy. Therefore, the *CSF*_ext_ can be used as a powerful tool for optimizing the fractionation conditions and making a decision on the selection of optimal operation parameters. 

FA fractionation generally produces three products for further processing, namely, cellulosic solid, hemicellulosic syrup and high-purity lignin. The hemicellulosic sugars obtained from sugarcane bagasse are primarily composed of pentose such as xylose, which can be converted to furfural under the catalysis of residual formic acid [7,30]. Moreover, after detoxification, pentose also can be converted to microbial lipid, a promising feedstock for biodiesel production [1]. High-purity lignin has relatively high added-value, but the down-stream products and market still need to be further developed. For cellulosic solids obtained by FA fractionation, since the cellulose contents were high, due to the removal of hemicelluloses and lignin, they can be used for the production of cellulose-derived materials. The cellulosic solid also can be hydrolyzed by cellulase enzymes to produce fermentable sugars. However, formylation takes place via the esterification of cellulose hydroxyl groups, which leads to a decrease in the cellulose digestibility. The degree of formylation is largely dependent on the reaction severity, especially formic acid concentration. Therefore, cellulose formylation should be considered for the selection of suitable *CSF*_ext_ when FA fractionation aims to increase the cellulose digestibility. Deformylation with bases such as ammonia, lime, etc., is necessary in order to recover the enzymatic hydrolysability of pretreated cellulosic solids. However, further investigation should be carried out, with consideration of the whole process design, and optimization from the perspective of techno-economics. 

## 4. Materials and Methods

### 4.1. Lignocellulosic Biomass and Chemicals

Sugarcane bagasse used in the experiments was obtained from the Guangxi Zhuang Autonomous Region in South China. It was air-dried and screened by sieves. The portion that could not pass through 40-mesh sieve was collected for FA fractionation. The main components of the bagasse were determined to be 43.4 ± 1.0% glucan, 24.4 ± 0.6% xylan, 1.2 ± 0.1% arabinose, 2.51 ± 0.03% acetyl group, 24.2 ± 0.6% klason lignin and 2.88 ± 0.06% acid-soluble lignin. Standard compounds used for HPLC calibration, including glucose, xylose, arabinose, and cellubiose were purchased from Sigma Aldrich (Shanghai branch, Shanghai, China).The cellulase (Cellic^®^ CTec2) used for the hydrolysis of pretreated substrates was kindly provided by Novozymes (Beijing branch, Beijing, China), which was a multi-enzyme formulation with a determined cellulase activity (filter paper activity, FPA) of 114.07 FPU/mL.

### 4.2. Formic Acid Fractionation of Sugarcane Bagasse

FA fractionation was carried out in a 500 mL three-neck glass flask heated by water bath or electric jacket under atmospheric pressure, with one of the necks connecting with a condenser. In this work, the liquid-to-solid ratio was selected as 15:1 (L/kg) in order to achieve a good system mixing. A total of 10 g of the screened bagasse was put into the three-neck glass flask, followed by the addition of 150 mL 60–90 wt% FA solution. Electrical stirring with a Teflon paddle was used at 300 rpm to keep the system as homogeneous as possible. The reaction temperature was controlled at 80 °C to the atmospheric boiling point of the aqueous FA solution (~107 °C) by a water bath or electric-heating jacket. The reaction time of the fractionation was in the range 0.25–2.0 h. After the reaction was finished, solids were recovered by suction filtration. The obtained solid was first washed with 150 mL 60–90 wt% FA solution to dissolve the residual lignin retained in the cellulosic solid and then filtered under pressure to remove as much liquid as possible. The solid was then rinsed with running water until neutrality. A portion of the washed solid was dried with acetone for chemical composition analysis, and the other part was stored at 4 °C for further enzymatic hydrolysis.

### 4.3. Enzymatic Hydrolysis of Pretreated Sugarcane Bagasse

The cellulosic solid obtained by FA fractionation were incubated at 50 °C, 150 rpm in 50 mM sodium acetate buffer (pH 4.8) in an air-bath shaker. All experiments were performed in duplicate with 10 mL working volume at an initial solid consistency of 5% (g/mL) with cellulase loading of 15 FPU/g solid for 120 h. Enzymatic digestibility was characterized by enzymatic glucan conversion (EGC, %), defined as the percentage of glucan enzymatically converted to glucose.

### 4.4. Analytical Methods

The main chemical components of the used bagasse and pretreated solid were determined according to NREL’s Laboratory Analytical Procedure [31]. The monosaccharide (glucose, xylose and arabinose) concentrations were determined by Shimadzu (Tokyo, Japan) HPLC (LC-10AT) system quipped with an Aminex HPX-87H column (BioRad, Hercules, CA, USA) and a differential refraction detector with 5 mM H2SO4 as an eluent at a flow rate of 0.8 mL/min. The kinetic parameters were fitted by nonlinear fitting tools of Microsoft Excel 2010 software (Redmond, WA, USA). 

## 5. Conclusions

An extended combined severity factor (*CSF*_ext_) was employed as an integrated parameter by involving the contribution of FA concentration, temperature and time to the reaction severity for modeling of the FA fractionation of sugarcane bagasse. Two phenomenological models based on the Arrhenius equation and modified Logistic equation were further used to describe the phenomenological kinetics, respectively. Different data-processing methods were compared to fit the severity parameters and model constants. It was found that using the data of solubilization of total xylan plus lignin fraction for fitting could obtain a good degree of fitting. Both Arrhenius-based and Logistic-based models showed a satisfying fitting accuracy for prediction of experimental data of xylan, lignin and xylan plus lignin solubilization, indicating that the linear models could generally be used to describe the phenomenological relationship between the solubilization of biomass components and *CSF*_ext_. Wide ranges of xylan solubilization (0.4–0.95), delignification (0.35–0.95) and total biomass solubilization (0.25–0.65) were observed, with an A-*CSF*_ext_ range of 26–46 or L-*CSF*_ext_ range of 37–47. Two distinct stages could be observed, as revealed by plots of *CSF*_ext_ v.s. xylan and lignin solubilization with inflection points at A-*CSF*_ext_ of 42 or L-*CSF*_ext_ at 43. For the enzymatic hydrolysis of cellulosic solids obtained by FA fractionation, the highest initial EGC (EGC@6h) was obtained at A-*CSF*_ext_ of 39–40 or A-*CSF*_ext_ of 40–41; however, for a relatively period of hydrolysis (EGC@6h), relatively high glucan conversion was observed at an A-*CSF*_ext_ of 42–43 or A-*CSF*_ext_ of 43–44. A higher *CSF*_ext_ might result in a higher degree of cellulose formylation, especially with high FA concentration, which conversely decreased cellulose digestibility. However, post-alkaline saponification with a small amount of lime could recover the cellulose digestibility. *CSF*_ext_ could be applied as an integrated parameter to evaluate the phenomenological kinetics of FA fractionation of sugarcane bagasse.

## Figures and Tables

**Table 1:** Determination of severity constants and model parameters by multiple linear regressions of experimental data under different conditions with different data-processing methods.

**Model based on Arrhenius equation**	**Data Used for Fitting**	**Fitted Severity Parameters and Kinetic Constants**						
		***ω***	***m***	***a***	***b***	***R*** **^2^**	***F* Value**	***p* Value**
	Xylan solubilization	22.53	8.23	0.1632	−4.30	0.8337	127.02	0
	Lignin solubilization	11.06	16.10	0.1439	−7.41	0.9076	295.74	0
	Xyan plus lignin solubilizaiton	0.1446	12.10	14.54	−5.5595	0.9142	269.84	0
	Total biomass solubilization	0.1193	8.9665	78.4760	−4.1200	0.5960	37.38	0
**Model based on Logistic equation**	**Data Used for Fitting**	***ω***	***m***	***q***	***c***	***R*** **^2^**	***F* Value**	***p* Value**
	Xylan solubilization	22.24	8.51	0.3093	−7.88	0.8496	143.06	0
	Lignin solubilization	10.40	16.83	0.2459	−12.60	0.9152	143.06	0
	Xyan plus lignin solubilizaiton	0.2640	12.52	13.91	−9.9289	0.9210	295.26	0
	Total biomass solubilization	0.1536	9.1787	87.6569	−4.9242	0.6003	38.05	0

**Table 2:** Orthogonal experiments and results of enzymatic digestion of bagasse pretreated with formic acid.

No.	*T* (°C)	*C*_FA_ (%)	*t* (h)	A-*CSF*_ext_	L-*CSF*_ext_	Experimental Results								
						SY (%)	GC (%)	XC (%)	LC (%)	XR (%)	DD (%)	FC (%)	EGC @6h (%)	EGC @72h (%)
1	80	60	0.25	35.95	37.11	68.7	56.17 ± 0.19	17.93 ± 0.09	21.16 ± 0.05	54.5 ± 0.3	39.06 ± 0.09	1.3 ± 0.03	4.1 ± 0.1	11.6 ± 0.1
2	80	70	0.5	38.75	39.97	64.7	63.7 ± 0.2	14.2 ± 0.2	18.9 ± 0.4	66.2 ± 0.5	48.6 ± 0.8	2.3 ± 0.01	6.0 ± 0.2	22.3 ± 2.5
3	80	80	1	41.27	42.56	51.6	74.2 ± 0.8	10.9 ± 0.06	13.6 ± 0.3	79.4 ± 0.1	70.7 ± 0.5	3.9 ± 0.02	4.8 ± 0.6	29.2 ± 0.4
4	80	90	1.5	43.29	44.63	43.4	77.9 ± 0.3	6.3 ± 0.05	6.7 ± 0.2	89.9 ± 0.1	88.3 ± 0.4	4.7 ± 0.04	3.2 ± 0.1	36.0 ± 0.2
5	90	60	0.5	37.33	38.52	64.7	52.4 ± 1.2	17.4 ± 0.3	19.4 ± 0.02	58.4 ± 0.8	47.5 ± 0.1	1.7 ± 0.02	10.4 ± 0.2	35.5 ± 3.3
6	90	70	0.25	38.74	40.00	79.1	60.4 ± 1.6	13.4 ± 0.02	13.9 ± 0.7	60.9 ± 0.1	54.6 ± 2.4	2.1 ± 0.05	10.7 ± 0.4	42.1 ± 3.8
7	90	80	1	41.96	43.28	49.6	64.6 ± 1.8	13.2 ± 0.4	10.7 ± 0.3	75.9 ± 0.6	77.9 ± 0.7	3.6 ± 0.05	8.0 ± 0.5	48.6 ± 2.2
8	90	90	1.5	43.97	45.35	45.9	71.6 ± 1.6	9.9 ± 0.01	5.9 ± 0.7	90.8 ± 0.01	89.2 ± 1.4	5.2 ± 0.1	3.9 ± 0.3	42.3 ± 2.9
9	99	60	1	38.64	39.86	63.0	69.4 ± 0.1	11.5 ± 0.06	17.3 ± 0.1	70.2 ± 0.1	58.3 ± 0.2	1.93 ± 0.01	8.6 ± 0.2	40.8 ± 1.1
10	99	70	1.5	41.15	42.45	46.0	74.5 ± 0.5	9.1 ± 0.09	10.8 ± 1.1	82.8 ± 0.2	81.5 ± 2.0	2.0 ± 0.02	6.1 ± 0.3	44.6 ± 0.2
11	99	80	0.25	41.19	42.54	46.1	73.7 ± 0.05	10.9 ± 0.2	11.8 ± 0.1	79.4 ± 0.4	79.8 ± 0.3	2.23 ± 0.001	6.2 ± 0.1	44.4 ± 2.1
12	99	90	0.5	32.28	44.90	54.2	81.5 ± 1.2	6.5 ± 0.1	7.3 ± 0.2	85.5 ± 0.2	85.5 ± 0.4	2.39 ± 0.2	4.9 ± 0.5	49.8 ± 1.4
13	105	60	1.5	39.46	40.70	63.0	65.9 ± 1.5	10.2 ± 0.2	18.0 ± 0.4	76.2 ± 0.5	51.6 ± 1.2	1.7 ± 0.04	12.2 ± 0.2	44.8 ± 0.6
14	105	70	1	41.16	42.46	53.6	69.7 ± 0.5	10.8 ± 0.02	13.1 ± 0.1	78.7 ± 0.04	70.5 ± 0.2	2.4 ± 0.02	7.9 ± 0.73	43.8 ± 6.8
15	105	80	0.5	42.30	43.66	46.7	83.0 ± 1.2	7.9 ± 0.1	7.6 ± 0.1	84.8 ± 0.2	87.1 ± 0.2	2.42 ± 0.3	5.9 ± 0.5	54.1 ± 0.5
16	105	90	0.25	43.21	44.64	46.7	81.4 ± 0.1	7.0 ± 0.03	6.7 ± 0.1	86.6 ± 0.1	88.7 ± 0.1	2.28 ± 0.001	5.4 ± 0.4	53.4 ± 1.0

**Table 3:** Analysis and enzymatic digestion of lime-deformylated solids.

De-formylated Solid	Experimental Results							
	SY (%)	GC (%)	XC (%)	LC (%)	DD (%)	FC (%)	EGC@6h (%)	EGC@72h (%)
Cellulosic solid obtained with 80% FA, 105 °C, 0.5 h	55.0	80.2 ± 8.0	9.7 ± 0.9	9.3 ± 0.09	78.8 ± 0.2	0.22 ± 0.01	43.7 ± 1.6	72.1 ± 1.4
Cellulosic solid obtained with 90% FA, 105 °C, 0.25 h	53.1	82.7 ± 4.5	9.3 ± 0.5	8.0 ± 0.1	82.4 ± 0.3	0.39 ± 0.03	40.9 ± 1.8	74.9 ± 0.3

## Data Availability

Not applicable.

## Sample Availability

Samples of the fractionated cellulosic pulps are available from the authors.
